# Microplastics in the Atmosphere and Water Bodies of Coastal Agglomerations: A Mini-Review

**DOI:** 10.3390/ijerph20032466

**Published:** 2023-01-30

**Authors:** Mengrong Bao, Xiaoqin Xiang, Jianshi Huang, Lingwei Kong, Juan Wu, Shuiping Cheng

**Affiliations:** 1 Institute of Eco-Environmental Engineering, College of Environmental Science and Engineering, Tongji University, Shanghai 200092, China; 2 Key Laboratory of Coastal Environment and Resources of Zhejiang Province, School of Engineering, Westlake University, Hangzhou 310024, China; 3 Shanghai Institute of Pollution Control and Ecological Security, Shanghai 200092, China

**Keywords:** atmospheric microplastic, freshwater, seawater, urban city, transport, health threat

## Abstract

Microplastics are ubiquitously in various environments from the equator to the poles. Coastal agglomerations act as both a source and sink connecting the global microplastic cycles of oceans and continents. While the problem of microplastics is particularly severe and complex in the coastal zones, where both inland and marine pollution are concentrated, the present study aimed to provide hot topics and trends of coastal urban microplastic studies and to review the researches on microplastic pollution in the atmosphere and water bodies in coastal agglomerations in terms of characteristics, behavior, and health threat of microplastics. The results of the bibliometric analysis showed an increase in the annual output of microplastic research. Research hot topics and clusters were analyzed using the VOSviewer. Characteristics of microplastics varied in abundance, size, and polymer type in different environments and countries. Furthermore, coastal cities are taken as a system to sort out the input, output, and internal transmission pathways of microplastics. The health threat of microplastics to urban residents was briefly reviewed and the exposure and health risks of microplastics to infants and young children were of particular concern. Detailed and comprehensive studies on intervention and reduction in the transmission of microplastics between the atmosphere and water bodies, whether microplastics are harmful to infants and young children, and measures to reduce the risk of microplastic exposure are needed.

## 1. Introduction

Microplastic (MP) as a term mostly refers to plastic particles smaller than 5 mm in size, thus ranging from milli-, micro-, to nano-dimension. Marine and freshwater microplastics (MPs) have been the focus of previous studies. MPs have been detected in rivers, estuaries, and inshore and pelagic waters [1,2,3,4], including polar seawaters [5,6], posing a non-negligible ecological threat to aquatic plants and animals [7,8,9]. The COVID-19 pandemic in recent years has increased the use of personal protective plastic products, resulting in the production of plastics, such as face masks that are carelessly discarded in the environment, which has contributed to MP pollution [10]. Since 2015, the focus of MP research has expanded from water bodies to the atmosphere. Previous studies have shown that the distribution of atmospheric MPs has spread all over the world and is one of the most difficult environmental challenges facing contemporary society [11,12,13]. MPs in the atmosphere have been reported in remote, inhospitable, and inaccessible areas, such as the Pyrenees in France [14], a Western nature reserve in the United States [15], and the Arctic archipelago of Svalbard [16]. The Norwegian Institute of Atmospheric Research conducted global simulations of the atmospheric transport of MP particles from global road traffic and found that the transport of these plastic particles to remote areas is highly efficient, with about 30% settling in the global oceans through this route [12]. This further quantifies the atmospheric transport and deposition of MPs and highlights the transport behavior of atmospheric MPs as a topic worthy of more research efforts.

As an active place for human production, living, and recreation, the city is an important source and sink of MPs in the atmosphere and water. However, most of the existing research focuses on the monitoring and investigation of MP point source pollution (such as sewage treatment plants and factory soil) [17,18] and water non-point source MP pollution (such as surface runoff) [19], and few studies on the generation, transmission, and occurrence of MPs from the perspective of the urban system. In particular, urban agglomerations (an extended city or town area comprising the built-up area of a central place and any suburbs linked by continuous urban area) close to the coastline are located at the ocean and mainland junction, which are not only polluted by inland, marine, and internal MPs but also have a complex internal transmission relationship. Therefore, the role of coastal agglomerations in global MP pollution is worth studying.

The objectives of this study were first, to analyze the hot spots and trends of related research based on bibliometric statistics, and second, to review the characteristics, sources, sinks, and internal transmission pathways of MPs in coastal urban agglomerations from the perspective of the urban system, as well as the health risks to the urban population.

## 2. Data Source and Methodology

The literature data in this article were gathered from the Web of Science (WoS) database. Advanced search mode was used to retrieve a total of 275 pieces of literature that match the theme of “MPs in coastal cities”. The complete search formula (and the number of retrieved literature) was as follows:
#1 (TS = (microplastic*)) AND TS = (coast*)(1844 articles)#2 (TS = (microplastic*)) AND (TS = (city*) OR TS = (urban*))(937 articles)#1 AND #2(275 articles)

There were five main document types of candidate literature set: Articles, review articles, meetings, early access, and books, of which articles accounted for the highest proportion (252, 91.6%), followed by review articles (19, 6.9%).

These 275 publications were then analyzed in the following two aspects: The distribution and trend of annual publication output and the co-occurrence analysis of keywords. The search was conducted in May 2022, and due to the ongoing publications in 2022, the 25 articles published in 2022 were not counted when analyzing the annual output to avoid misevaluation of the trend of publications. The latter aspect was generated using VOSviewer (version 1.6.18, Centre for Science and Technology Studies, Leiden University, Leiden, The Netherlands) software, an open-source literature visualization software.

## 3. Results on Bibliometric Analysis

### 3.1. Publication Outputs and Trends

All 275 candidate publications retrieved from the WoS database mostly belonged to the research area of Environmental sciences ecology (236, 85.8%) and Marine freshwater biology (95, 34.5%).

**Figure 1:**
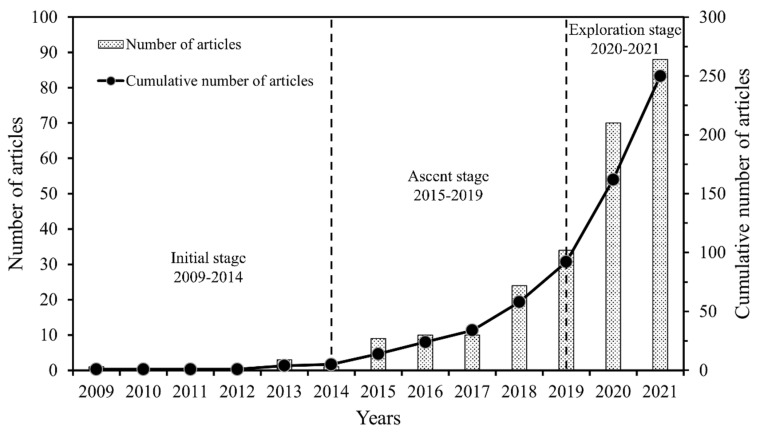
Annual and cumulative number of publications on microplastics in coastal agglomerations from January 2009 to December 2021.

The annual publication and a cumulative number of publications on MPs in coastal agglomerations are shown in Figure 1. The earliest article retrieved on WoS was published in 2009, proposing the phenomenon of the accumulation of mega- and macro-plastics near urban centers [20], after which no relevant research literature was retrieved until 2013. From 2013 to 2014, there was a slight increase in the number of publications (four in total). In 2015, several articles focused on MP pollution in coastal zones were published [21,22] and further suggested that coastal wastewater treatment plants (WWTPs) are potential point sources of marine MP pollution [23]. Since then, MPs in coastal agglomerations began to attract the attention of researchers, and the number of publications grew between 2015 and 2019. Then, came the years 2020 and 2021, wherein the number of published papers grew rapidly. Only in 2020, the number of publications was close to the sum of the previous years. Additionally, in 2021, 88 publications were achieved.

According to the growth rate of publications, we divided the research on MPs in coastal agglomerations into three stages: The initial stage from 2009 to 2014, the ascent stage from 2015 to 2019, and the exploration stage from 2020 to 2021 (publications in 2022 still remain unknown, thus are not included).

### 3.2. Research Hot Topics

**Figure 2:**
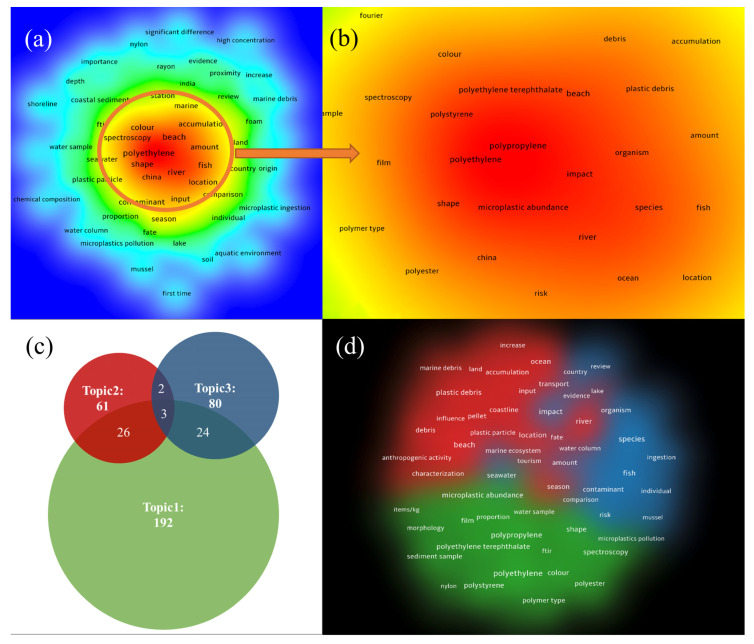
The item density and cluster density visualization map on microplastics in coastal agglomeration researches based on occurrences from January 2009 to May 2022. (**a**) The item density view. (**b**) The magnification of the high-occurrence area in Figure 2a. (**c**) Venn diagram of the literature number of three clusters. (**d**) The cluster density view. In the item density view, gradient colors are shown by red-blue-green according to the term occurrence, the closer to the red area represents the higher occurrence of the term. In the cluster density view, different colors represent different clusters divided (the same color corresponding to the clustering class in Figure 2c).

With the binary analysis mode of VOSviewer, a total of 7235 keywords (or phrases) were generated in the 275 publications obtained before. Then, the minimum frequency of keyword occurrence was set to 10 times, and 170 terms met the threshold. Thereafter, according to the default setting, 60% of the most relevant terms (102 terms) were selected for visualization. During this process, different terms with the same meaning were merged, such as “microplastic pollution” and “microplastic contamination”, “PVC” and “polyvinyl chloride”. Meaningless terms, such as “knowledge”, “current study”, and “data” were eliminated. Finally, 88 terms were obtained, and a set of density and clustering maps were further drawn (Figure 2), including a Venn diagram of the literature number of three clusters screened by manual classification (Figure 2c).

As seen in Figure 2b (the magnification of the high-occurrence area in Figure 2a), terms, such as “polyethylene”, “polyethylene terephthalate”, “polypropylene”, and “polystyrene” were of high item density, which indicated that these polymer types were hot topics in these researches. In addition, terms, such as “beach”, “river”, and “China” showed the hot research location. Additionally, terms, such as “microplastic abundance”, “shape”, “color”, “species”, and “fish” presented a general characterization of MPs (including biological indicators) in coastal agglomerations.

Three major term clusters (small clusters had been merged) of research on MPs in coastal agglomerations were generated with the binary analysis mode of VOSviewer: (1) MP abundance, shape, color, polymer type, and detective method in water and sediment samples, etc. (Figure 2c,d, green part,192 articles); (2) accumulation, transport, influence, and fate of MPs in the ocean, coastline, land, and river, etc. (Figure 2c,d, red part, 61 articles); (3) impact and health risks of MPs in organisms, including mussel, fish, and human, etc. (Figure 2c,d, blue part, 80 articles). According to the number of published papers (Figure 2c), most of the current studies focus on the physical and chemical characterization of MPs in urban environments, while their environmental behaviors and ecological risks are slightly weak.

It is worth mentioning that although the terms related to atmospheric MPs did not appear in both density views, the role of atmospheric MPs has become increasingly important according to recent research [11,24], which showed that the knowledge of coastal agglomerations airborne MPs is lacking (only 13 articles are related in the 275 publications obtained), and further additional research is needed in the future.

The characteristics, behaviors, and ecological risks of MPs in the urban environment will be reviewed in the following sections according to the clustering classification of keywords.

## 4. Characteristics of MPs in Coastal Agglomerations

Sampling and monitoring of urban freshwater systems, estuaries, surface and deep seawater, surface runoff, and influent and effluent of WWTPs have been reported more frequently around the world, and although data are still insufficient, this is a fundamental part of the background research on MPs in coastal agglomerations. MPs in different coastal urban waters vary in abundance and characteristics. Among them, MP pollution tends to be more severe in small freshwater bodies than in estuaries and coastal areas [25]. In addition, WWTPs have become a research hotspot as a point source [23] for MPs in urban water systems and coastal environments. The removal efficiency ranged from 70%–98%, while the size, shape, and type of MPs affected the removal and were related to SS concentration in the influent water and operational load [18,26,27,28].

To date, there have been far more studies of MPs in the aqueous environment than in the urban atmosphere. Among them, several reviews on MPs in urban and coastal waters have been described [29,30,31]; therefore, we will not repeat herein.

Atmospheric MPs are more time-sensitive and transportable than those in the aqueous environment, and studies have shown that atmospheric MP deposition during cooking and eating is more significant than by direct dietary intake [32]. Therefore, urban atmospheric MPs deserve more attention. Based on the literature set searched as previously mentioned, the abundance and characteristics of MPs reported in the few published urban atmospheric MPs literature were summarized in Table 1. 

Atmospheric microplastics sampling methods are broadly divided into active and passive sampling, the former is usually used to collect suspended aerosols using pump-powered total suspended particulate samplers, and the unit of microplastics is often expressed as particle/m^3^ or n/m^3^, while the latter usually uses non-plastic containers with funnels to collect atmospheric deposited particulate matter under the action of gravity, and the unit is often expressed as settling flux n/(m^2^·d). Occasionally, n/g is also used to indicate microplastic deposition, when the collected sample is usually dust or soil. After pre-treatments, such as sieving, ablation, density separation, and filtration, microscopy and electron microscopy are commonly used to observe and characterize the physical properties of microplastics (e.g., color, morphology, length, degree of wear, etc.), and further to determine the chemical characteristics of microplastics (polymer type, additive composition, etc.) using FTIR, Raman spectroscopy, SEM-DES, LC-MS/MS, etc.

The levels of MPs in the urban atmospheric environment in the available studies showed that the units of measurement are not uniform in the presentation of study data. In terms of deposition fluxes, the highest deposition levels were found in Yantai, China (130–624 n/m^2^·d) while the lowest was in Jakarta (3–40 n/m^2^·d). Studies in China focused on coastal cities (Shanghai, Yantai, Dongguan), which is important for understanding the sea-land transport of atmospheric MPs. Fibers were the dominant form of MPs in the above cities (except for Wenzhou, China, and Hamburg, Germany, where most of the atmospheric MPs are fragments). Klein and Fischer [33] speculated that this difference was likely due to the different methods used in the study, including sample size, sampler height, and analysis methods. Some debris, films, and foam were detected in atmospheric deposition in Dongguan, Shanghai, Yantai, and Jakarta. Due to the difficulty of manually picking out microplastics and the limitations of the minimum detection limits of spectroscopy-type detection machines, such as FTIR, the minimum size of MPs varies from 2 to 200 μm, although there is a trend to lower the detection limit to 50 μm after 2019. However, the trend of the particle size distribution shows that the number of MPs increases with the decreasing particle size, thus the presence of a large number of small particle-size MPs in the air may be more hazardous to human health [14,34,45]. Polymer identification assists in analyzing the source of MPs. In most studies of atmospheric MPs, the chemical composition of MPs was identified. It can be concluded that polypropylene (PP), polyethylene (PE), polystyrene (PS), and polyethylene terephthalate (PET) were the main polymer components in atmospheric deposition. Some characteristic MPs can reflect the specificity of pollution at the sampling site, for example, PB (polybutadiene) was only present in atmospheric deposition in Jakarta compared to the atmosphere of other cities [35]. This assists in tracing the specific sources of MPs contamination, which can be controlled and eliminated at the source. Moreover, the backward trajectory is a common way to trace the sources of atmospheric MPs. Jia et al. [46] found that airborne MPs in Shanghai, a megacity, are dominated mainly by exogenous sources, and that precipitation and wind were the main influences on atmospheric MP deposition fluxes and characteristics at small scales, while at large scales, the fluxes were closely related to population size. 

## 5. Behaviors of MPs in Coastal Agglomerations

**Figure 3:**
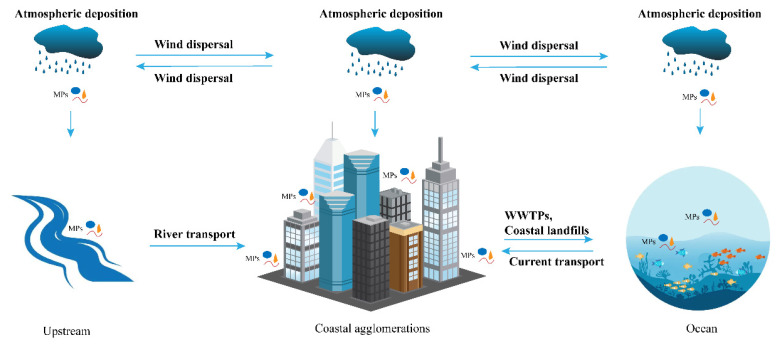
Microplastic input and output pathways in coastal agglomerations (symbols from the IAN/UMCES Symbol and Image Libraries were used).

The behaviors of MPs in coastal agglomerations contain intricate material exchanges among the sea, the interior, and the participating cities. As a result, the urban agglomerations are considered a system (i.e., a coastal urban system) in this research, which allows for a simplified and clear discussion of their MP input and output as well as the specific types of MP sources, sinks, and transmission pathways within (Figure 3).

### 5.1. MP Input in the Coastal Urban System

The current research shows that the input of MPs in coastal agglomerations mainly includes atmospheric transport, upstream freshwater input, and marine current input. Since this paper mainly reviews behaviors in the atmosphere and water bodies, the behaviors of soil MPs will not be elaborated.

Marine MPs can be transported into coastal cities by atmospheric pathways. MPs in seawater will be aerosolized into the atmosphere due to the action of wind or ocean waves, thus entering the transmission system of the atmosphere, being input to coastal cities by the action of wind, and entering the urban system through the process of dry or wet deposition. A regional atmospheric transport model established by Long et al. calculated that the adjacent Atlantic and Indian Oceans inputted approximately 0.35 kt of MPs larger than 10 μm per year to the Asian continent [47]. Similarly, inland MPs could also be transported to coastal cities due to meteorological factors, such as monsoons [48].

The second input is the transmission through the river. MPs with a density lower than water in the upstream freshwater are easy to migrate in the river through water flow, while high-density MPs are easy to settle in the short-range migration and occur in the sediments of downstream urban rivers. Moreover, the migration of MPs is affected by hydrodynamic conditions, weather conditions, MP size, morphology, biofilm attachment, etc. [49]. Luo et al. monitored the abundance of MPs (1.8~2.4 items/L) in freshwater, such as urban streams (Shanghai), Suzhou River, Huangpu River, estuarine, and near-coastal water bodies (0.9 items/L), indicating that the pollution of MPs in urban rivers was more serious than in estuaries and near-coastal water bodies, and more than 80% of MPs in urban rivers were found to be fibers [25], which had the possibility of long-distance transmission from upstream. In addition, studies had demonstrated the impact of flood events on the transport of MPs, with river MP concentrations increasing 5–8 folds during floods, especially more significantly at downstream sites [50].

The input of ocean waves could not be neglected. Marine-derived MPs (such as fishing gear fibers and ship paint debris) and nearshore MPs (such as coastal textile industry outflows) are carried by wind and waves and can be deposited and stored in coastal zones. This deposition process was not only influenced by the physical properties of the MPs themselves (size, morphology, density) but was also largely controlled by storm surge events [51].

### 5.2. MP Output in the Coastal Urban System

Coastal agglomerations may continue to pollute the ocean with MPs through atmospheric transport. The concentration of suspended MPs in the atmosphere in coastal areas is higher than the ocean, which makes the MPs in coastal cities migrate to the sea, especially textile fiber MPs, which are easily transported through the atmosphere. Liu et al. estimated that 1.21 t of suspended MPs entered the marine ecosystem of the western Pacific per year [52]. Furthermore, taking the size and morphology of MPs into consideration, Long et al. estimated that the Asian continent exports about 4.2 kt of MPs to the adjacent Atlantic and Indian Oceans per year through a model [47].

MPs in coastal cities may also import MP pollution inland through atmospheric transport. Several studies have found the appearance and deposition of atmospheric MPs in sparsely populated polar regions and the Tibet Plateau [16,53], confirming the existence of long-distance transmission of atmospheric MPs. The source of atmospheric MPs could be roughly estimated based on wind strength, wind direction, and particle size. Atmospheric MP monitoring in south-central Canada revealed that potential sources of MPs included Toronto (200 km away) [54].

In addition, coastal cities can export MPs to the ocean through urban WWTPs and coastal landfills. According to research surveys, a WWTP in northern France was estimated to discharge 227 million MPs particles per day, and high concentrations of MPs had been observed in water bodies near coastal landfills, indicating that they served as an important route for MPs to enter the marine coastal environment [26].

### 5.3. MP Sources and Sinks in the Coastal Urban System

**Figure 4:**
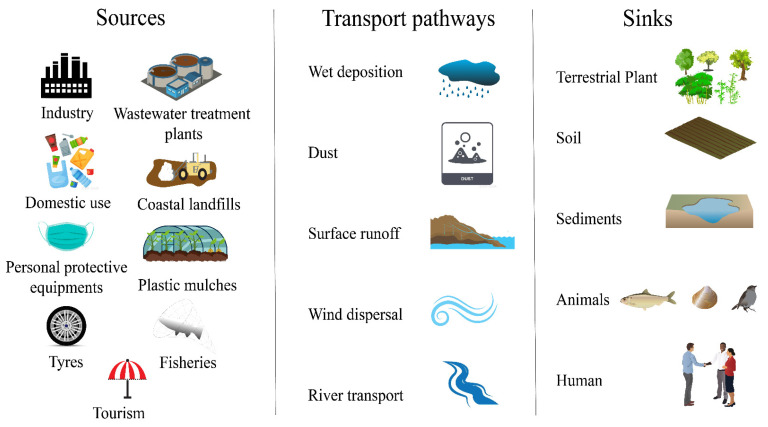
Microplastic sources, transport pathways, and sinks in coastal agglomerations (symbols from the IAN/UMCES Symbol and Image Libraries were used).

There are complex MP transport networks within coastal urban systems. Figure 4 is an overview of the source, sink, and intermediate transmission pathways. This summarization assists in controlling MP pollution at the source and the end, and hinders the transmission of MPs through controllable engineering and technical means, thereby reducing its ecological risk.

The main sources of MPs in coastal agglomeration systems are industrial production (such as textile factories, production of plastic microbead sprays for derusting ships), domestic plastic uses (plastic bottles, plastic bags, etc.), personal protective equipment (such as masks frequently used during the COVID-19 pandemic), tires, agricultural mulches, coastal landfills, fisheries, tourism, domestic sewage, coatings and paints for buildings. In particular, protective masks, which have surged in per capita usage during the epidemic, are an important source of MPs in urban systems. Most of the MPs released from masks are medium-sized transparent polypropylene fibers from non-woven fabrics. Wear and aging of masks could cause an increase in meso-sized blue MPs, thus enhancing the release of MPs, and masks may also enrich airborne MPs during use [55].

The sinks of MPs in the coastal urban system are mainly sediments, and organisms (may pose a health risk to humans through the food chain). Sediments, such as riverbed sediments, mangrove sediments, beaches, and wetland sediments in coastal urban systems, are the main sinks of MPs, and benthic organisms (such as hard corals) with complex structures and rough surfaces have higher MP capture rates [56]. Moreover, due to the small particle size of MPs, which is similar to algae and zooplankton, they are easy to be mis-ingested by marine organisms and enter into the food chain, resulting in a series of ecological toxic effects (inhibition of growth and development, the influence of feeding and behavior ability, reproductive toxicity, immune toxicity, genetic damage, etc.) [57]. In a survey of biological samples of reef fish from the southwestern Atlantic Ocean, ingestion of clear particles dominated, with polyamide as the most common plastic material, while domestic sewage, fishing activities, and navigation appeared to be the main sources of microplastic ingestion by reef fish [58].

It is worth mentioning that some media play both the roles of source and sink, such as terrestrial plants, soil, and urban WWTPs. Atmospheric MPs could attach to plant leaves during their transport, but could also resuspend into the atmosphere by air disturbances [59]. In terrestrial environments, soils are the boundaries of different environmental compartments, and when MPs enter the soil, they can accumulate and reach high concentration levels, affecting soil biodiversity and potentially causing phytotoxicity [60]. Similarly in WWTPs, most of the MPs will be removed in the sewage treatment process and enriched in the sludge, but some of them will enter the urban/marine water environment with the effluent.

The transmission pathways of MPs in coastal urban systems mainly include wet deposition, dust fall, surface runoff entrainment, wind transmission, and transport within urban river networks. Atmospheric MPs that have settled in terrestrial environments may be resuspended in the air or transported to aquatic systems by rainwater or surface runoff [42,61], where they are subsequently hosted in sediments. Surface runoff is considered to be an important route for the transport of MPs from the terrestrial environment to the aquatic environment, while the presence of vegetation could effectively reduce the transmission rate of MPs [19]. The impact of riverine transport should not be underestimated, as Karthik et al. found that beaches adjacent to rivers showed a relatively high abundance of microplastics compared to beaches affected by tourism and fishing activities [62].

## 6. Health Threat of MPs to Human Beings

Previous studies have confirmed the widespread presence of MPs in the atmosphere, which poses exposure risks to the human body in both indoor and outdoor environments. In particular, people will be exposed to air-containing MPs for a long time during long-term home isolation and office hours during the COVID-19 pandemic. Studies have shown that indoor MPs have higher concentrations than outdoor environments [34]. Prata et al. speculated that humans may inhale 26 to 130 MP particles per day [63], and Liu et al. estimated that Shanghai citizens inhale about 21 MP particles per day from outdoor environments [64].

Human contact with MPs mainly includes inhalation (air) [45], ingestion (seafood, beverage, dust reduction, etc.) [65] and skin contact (cosmetics, etc.) [66]. MPs entering the human body may lead to oxidative stress, chromosomal translocations, inflammation, and particulate matter accumulation in the human body [67]. Some special populations, such as children and workers in the textile industry, may be significantly affected by MP pollution due to the high abundance of MPs near the ground and the lack of proper protection in the work area, respectively [45]. Previous studies had measured the mass concentration of MPs in the blood of subjects as 1.6 µg/mL, indicating that plastic particles can be absorbed into the human blood, and inferred that they are likely to come into contact (or ingested or inhaled) through mucous membranes [68]. The typical residence time and absorption model of MPs in the human body urgently need further research.

Chemical additives in MPs, particularly those used as retarders and/or plasticizers, have been well-documented to cause adverse effects on human health. These include endocrine disorders, reproductive toxicity, neurotoxicity, hepatotoxicity, and cancer [69], with evidence supporting the hypothesis that MPs and their additives are potential obesogens [70]. Due to the small MP particles size, large specific surface area, and the concerns of exposure to the toxic chemical additive added, these chemicals from plastic polymers have more possibility to infiltrate people’s body fluids (such as sweat, stomach, intestine, and lung fluid) which are exposed (i.e., the higher biological availability) [71].

**Figure 5:**
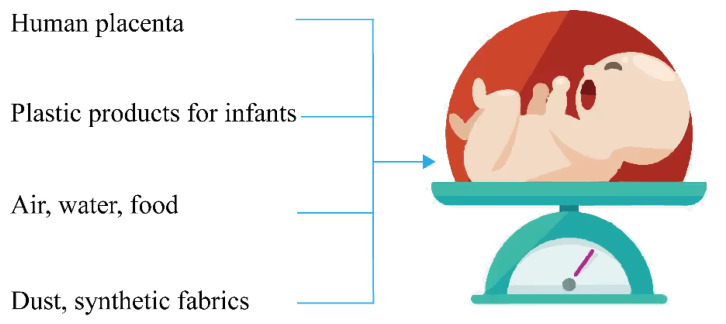
Pathways of microplastic threatening the health of infants and young children.

In particular, the dangers of MPs to infants and young children deserve more attention. Several pathways of microplastic can threaten the health of infants and young children (Figure 5). Biological surveillance studies of infant feces [72] and placenta [73] have provided direct evidence of MP exposure in infants and young children. Maternal transfer of MPs to the developing fetus has been demonstrated in exposed experimental animals [74]. The amount of MPs in the feces of infants is 10–20 times higher than adults [72], which may be due to the higher exposure of infants to plastics and air. Babies habitually nibble on bottles and are exposed to MPs when feeding formula. Li et al. found that each liter of heated liquid in polypropylene baby bottles can release 1.3 to 16 million MPs and trillions of smaller nanoplastics. According to the current guidelines for bottle disinfection and feeding formula preparation, infants are exposed to an average of more than 1 million MPs particles per day. Higher rates of bottle use and daily infant milk consumption are associated with a higher risk of MP exposure in developed countries with relatively lower breastfeeding rates than in developing countries [75]. Infants’ daily intake of MP fibers was significantly higher than the other age groups since infants spent more time indoors, where MP levels are often considerably higher than outdoors [76].

## 7. Conclusions and Prospects

Since 2009, research on MPs in coastal agglomerations have increased and can be divided into three main clusters: Characteristics, behaviors, and ecological risks of MPs. Despite the larger number of studies on MPs in urban waters, the atmospheric MPs are of great significance to the transmission process of MPs, and it is necessary to strengthen the research in this area. In addition, the monitoring and quantitative methods of atmospheric MPs need to be unified. Coastal cities as an important source, sink, and transmission carrier of MPs, complex combined transport processes, and the behavior of microplastics occur daily within and at the boundary of cities, thus further exploration of the transmission process and interception means of MPs in coastal agglomerations are required. In terms of ecological risk, people staying indoors for a long time tend to aggravate the harm of MPs to the human body due to the COVID-19 pandemic and other factors. Moreover, several pathways of MP are threatening the health of infants and young children. While their immune function is not yet complete, there is an urgent need to assess whether current levels of exposure to MPs pose a risk to infant health and to explore the formula preparation scheme in order to reduce the exposure of infants to MPs.

## Figures and Tables

**Table 1:** The prevalence of airborne microplastics in different urban regions.

Country/Region	The Concentration of Airborne MPs (n/ m^2^/d) *	Type of MPs	Size of MPs (μm)	Polymer Type	Reference
Edinburgh, UK	2420 ± 1597	fiber	100–5000	PET, PEU	[32]
Hamburg, Germany	76.5 ± 49.8 (open field)	fragment, fiber	63–5000	PE, EVAC, PTFE, PVA, PET	[33]
Paris, France	0.4–59.4 n/m^3^ (outdoor) 0.9 ± 0.6 n/m^3^ (indoor) 33% MPs	fiber	50–3250	PA, PP	[34]
Jakarta, Indonesia	3–40	fiber, fragment, foam	200–5000	PET, PS, PB, PE	[35]
Paris, France	29–280	fiber, fragment	100–5000	N/A	[36]
Paris, France	110 ± 96 (urban) 53 ± 38 (sub-urban) 29% MPs	fiber	50–5000	RY, PET, PA	[37]
Dongguan, China	36 ± 7	fiber, fragment, film, foam	0–4200	PE, PP, PS	[38]
Yantai, China	130–624	fiber, fragment, film, foam	50–3000	PET, PE, PVC, PS	[39]
Shanghai, China	0.27–1.33 n/m^3^	fiber, fragment	12–2191	PET, EP, PE, ALK	[40]
Shanghai, China	0–4.18 n/m^3^	fiber, fragment, granule	23–5000	PET, PE, PES, PAN, PAA, RY, EVA, EP, ALK	[41]
Asluyeh, Iran	60 n/g	fiber, film, fragment, spherule	2–5000	N/A	[42]
Asia-Pacific	23.04–67.54 (deposited atmospheric MPs) 0–1.37 (suspended atmospheric MPs)	N/A	16−4290	N/A	[43]
Wenzhou, China	1583 ± 1180 n/m^3^ (indoor air) 189 ± 85 n/m^3^ (outdoor air) 224 ± 70 n/m^3^ (urban areas) 101 ± 47 n/m^3^ (rural areas)	fragment, fiber	5–5000	PES, PA, PP, PE, PS	[44]

* The “n” in the unit of n/ m^2^/d refers to the number of MPs particles. Abbreviations: PET: Polyethylene terephthalate; PE: Polyethylene; PES: Polyester; PAN: Polyacrylonitrile; PAA: Poly(N-methyl acrylamide); RY: Rayon; EVA: Ethylene vinyl acetate; EP: Epoxy resin; ALK: Alkyd resin; PB: Polybutadiene; EVAC: Ethylene vinyl acetate copolymer; PTFE: Teflon; PVA: Polyvinyl acetate; PA: Polyamide; PS: Polystyrene; PE: Polyethylene; PVC: Polyvinyl chloride; PP: Polypropylene; PEU: Polyether urethane.
